# Maize Grain Metabolite Profiling by NMR: Effects of Growing Year, Variety, and Cropping System

**DOI:** 10.3390/molecules29174097

**Published:** 2024-08-29

**Authors:** Anatoly Petrovich Sobolev, Erica Acciaro, Milica Milutinović, Jelena Božunović, Neda Aničić, Danijela Mišić, Autar K. Mattoo

**Affiliations:** 1 Magnetic Resonance Laboratory “Annalaura Segre”, Institute for Biological Systems, National Research Council (CNR), Via Salaria km 29.300, 00015 Rome, Italy; 2 Institute for Biological Research “Siniša Stanković”-National Institute of Republic of Serbia, University of Belgrade, 11108 Belgrade, Serbia; milica.milutinovic@ibiss.bg.ac.rs (M.M.); jelena.boljevic@ibiss.bg.ac.rs (J.B.); neda.anicic@ibiss.bg.ac.rs (N.A.); dmisic@ibiss.bg.ac.rs (D.M.); 3 Beltsville Agricultural Research Center, Beltsville, MD 20705, USA

**Keywords:** *Zea mays*, cropping system, metabolomics, NMR

## Abstract

Considering that maize (*Zea mays* L.) is a staple food for a large segment of the population worldwide, many attempts have been made to improve the nutritional value of its grain and at the same time to achieve sustainable cropping systems. The present study aimed to characterize the composition and nutritional value of maize grain as influenced by cropping system, genetic background (variety), and growing year using untargeted NMR metabolomics. The composition of both water- (sugars and polyols, organic acids, and amino acids) and liposoluble metabolites (free and esterified fatty acids, sterols, and lipids) extracted from the maize grain was determined. Multivariate statistical analyses (PCA and ANOVA) pointed to the growing year and the variety as the most important random and fixed factors, respectively, influencing the metabolite profile. The samples were separated along PC1 and PC3 according to the growing year and the variety, respectively. A higher content of citric acid and diunsaturated fatty acids and a lower content of tyrosine, trigonelline, and monounsaturated fatty acids was observed in the organic with respect to the conventional variety. The effect of the cropping system was overwhelmed by the random effect of the growing year. The results provide novel knowledge on the influence of agronomic practices on maize micronutrient contents.

## 1. Introduction

Maize (*Zea mays* L.) is an important cereal crop, a staple food for a large segment of the population worldwide [1]. Maize kernels are considered a rich source of phytochemicals with nutritional (e.g., fatty acids, amino acids, proteins, carbohydrates, vitamins, fats, and minerals) and health benefits (e.g., carotenoids, phenolic compounds, and phytosterols) for humans. Even though maize kernels are often considered to be ‘nutrient-rich’ foods, which supply many macro- and micronutrients necessary for human metabolism, the amounts of some essential nutrients are insufficient for consumers that rely on maize as a major food source. Maize kernels lack two essential amino acids, viz. tryptophan and lysine, and are deficient in ascorbic acid (vitamin C), B vitamins, iron, and iodine [2,3,4].

It has been reported that yield, chemical composition, and nutritive value of maize grain are influenced by genetic background [5,6,7,8], environmental conditions [5,9,10], agronomic practice [11,12], and processing methods [13,14]. The agronomic practice has to face the challenges related to global warming in order to mitigate the impact of climate changes risks. Attempts have been made worldwide to improve the nutritional value of maize grain and to build crop resilience to abiotic and biotic stresses adopting conventional [15,16] and biotechnological [17,18] approaches, and at the same time to achieve sustainable cropping systems that have minimal or lesser impacts on the environment. Taking into account that crop genetics is the primary driver of plant nutrient content, it is extremely challenging to manage crop production fields and optimize crop nutrient content.

Equally important are attempts to develop and optimize analytical techniques for characterization of chemical composition and nutritional value of maize grain, to adequately interpret metabolomics data, as well as to develop quality control standards and methods and thus ensure food security and health benefits for humans. The term “metabolomics” is defined as the comprehensive and quantitative analysis of all small molecules in a biological system [19], and metabolomics analysis relies on appropriate analytical platforms, most commonly mass spectrometry (MS) and nuclear magnetic resonance (NMR) [20]. NMR spectroscopy is one of the most suitable analytical techniques employed for metabolomic studies [21], providing a comprehensive metabolite profile of samples, a robust quantification protocol, and spectroscopic/structural information on a wide range of metabolites with high analytical precision.

In our previous study we investigated cropping system impacts on maize grain nutritional content [22]. Grain was sampled from conventional and organic maize varieties grown for three growing seasons using five cropping systems. Different crop varieties were used in the conventional vs. the organic systems because the genetically modified crop varieties widely used by most conventional farmers are prohibited in certified organic systems. The five cropping systems included a three-year conventional no-till rotation (NT), a three-year conventional chisel-till rotation (CT), a two-year organic rotation (Org2), a three-year organic rotation (Org3), and a six-year organic rotation (Org6) [23]. These cropping systems represent a wide range of management practices used in the mid-Atlantic region of the US. They vary in the uses of legume and forage cover crops (such as hairy vetch, crimson clover (*Trifolium incarnatum* L.), and rye), tillage intensity (from full inversion tillage to reduced tillage and no-tillage), and fertility management, including materials used for nitrogen fertility. Metabolic fingerprinting of maize grain methanol extracts with ultra-high performance liquid chromatography (UHPLC) coupled with mass spectrometry (MS) was performed adopting both non-targeted and targeted approaches [22]. The composition of major methanol-soluble metabolites (e.g., phenolics and amides) was shown to be significantly influenced by the cropping systems, natural impacts (the growing year), and the maize cultivar genetics. The same experimental design (conventional vs. organic varieties, two growing seasons) although with a reduced number of cropping systems (only NT, CT, Org2, and Org3) was adopted in the present NMR-based metabolomics study. The use of the NMR metabolomics enabled us to extend the number of metabolites monitored and to further characterize the composition and nutritional value of maize grain as influenced by the cropping system. Here we describe the composition of both water- (sugars and polyols, organic acids, and amino acids) and liposoluble metabolites (free and esterified fatty acids, sterols, and lipids) and multivariate statistical analysis of metabolites variation as a function of random (the growing year) and fixed factors (the variety and the cropping system). This will provide novel knowledge on the influence of agronomic practices on maize micronutrient concentrations and contents.

## 2. Results

### 2.1. Water-Soluble Metabolites

#### 2.1.1. The Identification and NMR Spectral Assignment

Assignments of ^1^H NMR spectra of water-soluble maize grain extracts in D_2_O/phosphate buffer, Table 1, were based on 2D NMR experiments (^1^H-^1^H TOCSY, ^1^H-^13^C HSQC and ^1^H-^13^C HMBC) and a previous NMR study [24]. The twenty-two most abundant metabolites in the extracts were identified (6 organic acids, 3 sugars, 9 amino acids, and 4 miscellaneous metabolites).

#### 2.1.2. Metabolite Content

The main classes of water-soluble metabolites in the maize grain included sugars and polyols, organic acids, and amino acids (see Table 2 and Figure S1). Sugars were the most abundant metabolites, especially sucrose (up to 2 g/100g of DW). Among the nine amino acids quantified, proline was the most abundant, followed by asparagine and aspartate. Citric acid was the most abundant organic acid in the maize grain. Glycerol, choline, and glycerophosphorylcholine were also present. For comparison, Table 2 also shows the maximum and minimum content for all the metabolites, which indicates a noticeable variability for almost all of them, especially in the cases of malic acid, glucose, proline, and some other components. A more detailed picture of the variability and its sources was obtained by statistical analyses, discussed below.

### 2.2. Liposoluble Metabolites

#### 2.2.1. The Identification and Spectral Assignment

To the best of our knowledge, assignment of a ^1^H NMR spectrum of a maize grain extract in chloroform has not been reported in the literature. Identification of metabolites reported in Table 3 was based on 2D NMR experiments (^1^H-^1^H TOCSY, ^1^H-^13^C HSQC, and ^1^H-^13^C HMBC) and the literature on the NMR of corn oil [25] and complex lipid mixtures [26].

#### 2.2.2. Metabolite Content

The main components of the organic extracts were free and esterified fatty acids (see Table 4 and Figure S2). Four classes of fatty acids were distinguished: saturated (SFA), monounsaturated (MUFA), diunsaturated (DUFA), and polyunsaturated (PUFA). The fatty acid composition with a predominance of diunsaturated fatty acids (>50%) followed by monounsaturated and saturated fatty acids is consistent with the literature data on the composition of corn oil [27]. It was impossible to determine the content of individual fatty acids of the same class (e.g., palmitic acid and stearic acids among the saturated fatty acids) by ^1^H NMR analysis owing to complete overlapping of the corresponding NMR signals. Esterified fatty acids are the components of various lipids. Six different lipid classes were identified and quantified: triacylglycerides, 1,3-diacylglycerides, 1,2-diacylglycerides, 1-monoacylglycerides, 2-monoacylglycerides, and phosphatidylcholine. Triacylglycerides (TG) were predominant components of the lipid fraction. Diacylglycerides (1,2- and 1,3-) and 1- and 2-monoacylglycerides are intermediate products of TG hydrolysis. Complete hydrolysis of TG results in free fatty acids and glycerol production. A high percentage of free fatty acid (44% as a mean value) was present in the extracts, indicating a high degree of lipid hydrolysis. It is noteworthy that a good linear correlation (R^2^ = 0.89) between the free fatty acid content in the organic extracts and the glycerol content in the aqueous extracts was observed indicating that lipid hydrolysis was the main source of the glycerol and the free fatty acids in the corn grain samples. Moreover, three sterols were identified: campesterol, *β*-sitosterol, and stigmasterol. Unfortunately, all the ^1^H signals of campesterol were overlapped with those of *β*-sitosterol; therefore it was not possible to quantify them separately.

### 2.3. Effect of Variety, Cropping System, and Growing Year on Metabolite Profile

#### 2.3.1. PCA

**Figure 1:**
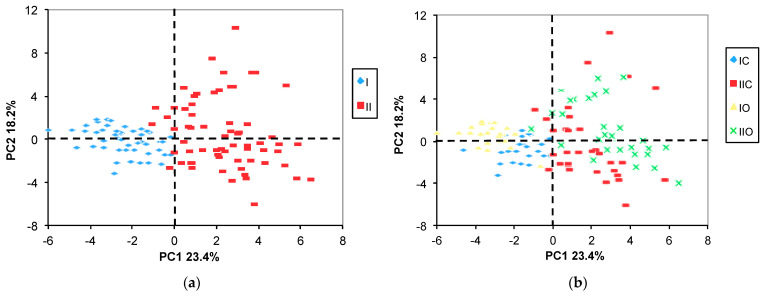
PC2 vs. PC1 score plots. The samples in (**a**) are labeled according to the year only, while in (**b**) the sample symbols indicate both year and variety. II and I correspond to 2nd and 1st year samples; O and C are organic and conventional varieties, respectively.

As an explorative unsupervised method, PCA was applied for the entire set of NMR data. The first four principal components accounted in total for 61.6% of the variability. As can be seen from the first score plot (PC2 vs. PC1, Figure 1a), the samples are separated along PC1 according to the growing year, indicating that the year-related metabolite variations were the highest in comparison to the other factors (variety or cropping system).

**Figure 2:**
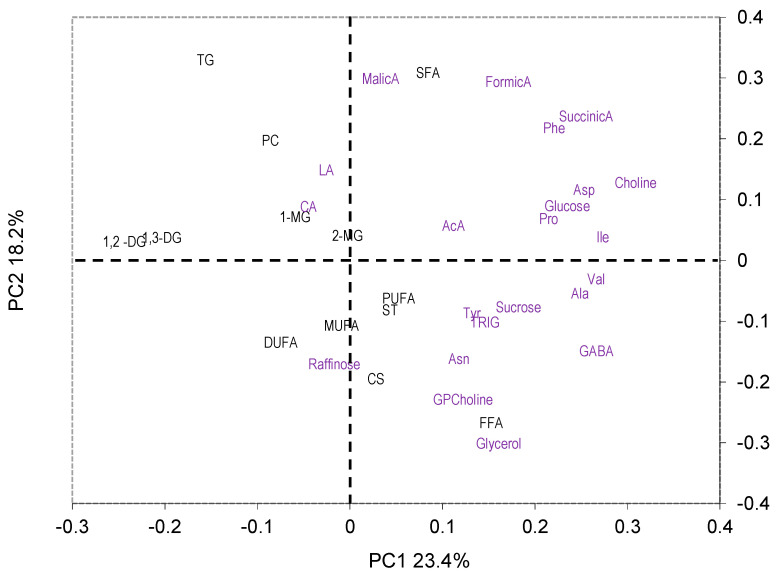
PC2 vs. PC1 loading plot. Abbreviations: AcA, acetic acid; CA, citric acid; LA, lactic acid; TRIG, trigonelline. Black and purple labels correspond to liposoluble and water-soluble metabolites, respectively.

The loading plot (Figure 2) indicates the metabolites with the highest variation between the two years (1,2-DG, 1,3-DG, choline, Ile, Val, GABA, Asp, Ala, succinic acid, glucose, Phe, and Pro) and suggests, in addition, that the levels of 1,2- and 1,3-diacylglycerols were the highest in the first year, whereas the levels of choline, Ile, Val, GABA Asp, Ala, succinic acid, glucose, Phe, and Pro were the highest in the second year.

**Figure 3:**
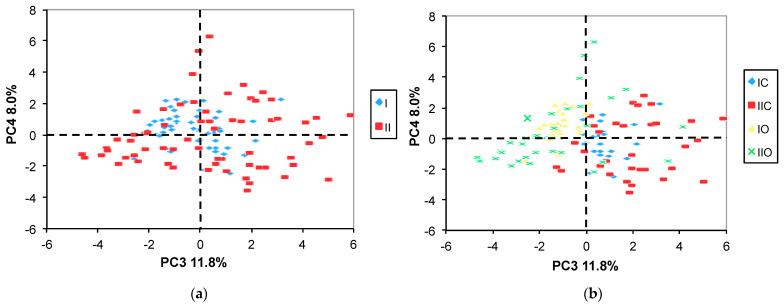
PC4 vs. PC3 score plots. The samples in (**a**) are labeled according to the year only, while in (**b**) the sample symbols indicate both year and variety. II and I correspond to 2nd and 1st year samples; O and C are organic and conventional varieties, respectively.

Moreover, the PC2 vs. PC1 score plot labeled for both the year and the variety (Figure 1b) evidences a partial separation of samples along the PC1 axes according to the variety (O vs. C). Additionally, a more evident separation between the O and the C varieties can be seen on the PC4 vs. PC3 score plot (Figure 3b).

**Figure 4:**
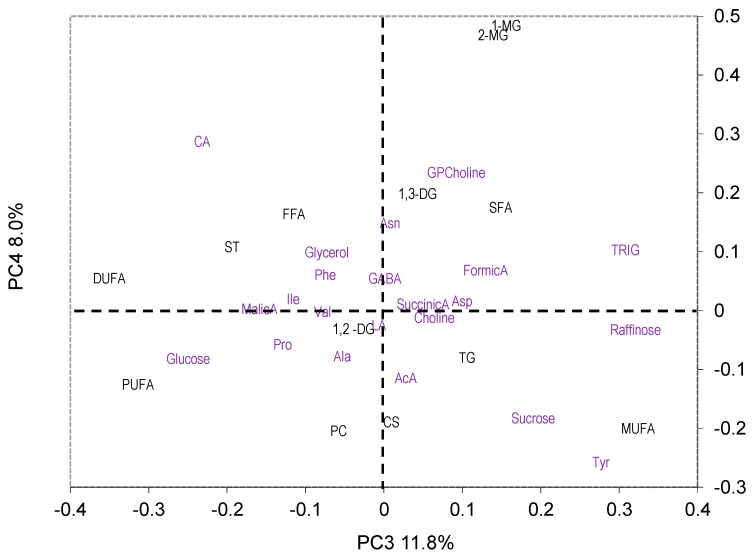
PC4 vs. PC3 loading plot. Abbreviations: AcA, acetic acid; CA, citric acid; LA, lactic acid; TRIG, trigonelline. Black and purple labels correspond to liposoluble and water-soluble metabolites, respectively.

**Figure 5:**
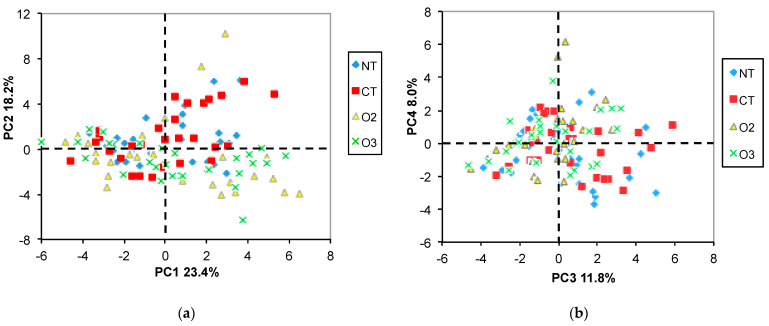
PCA score plots. (**a**) PC2 vs PC1; (**b**) PC4 vs. PC3. The samples are labeled according to the cropping system only.

As seen in Figure 3a, no year-related separation was present in the PC4 vs. PC3 plot, but the “O” samples are clearly separated from the “C” ones along the PC3 axis (Figure 3b). The loading plot (Figure 4) suggested that DUFA, PUFA, glucose, and citric acid (CA) were more abundant in the “O” samples, whereas MUFA, raffinose, trigonelline, Tyr, and sucrose were more abundant in the “C” samples. No clear separation according to the cropping system was observed (Figure 5a,b).

#### 2.3.2. ANOVA

Three-way ANOVA analysis (Y vs. V vs. S) was performed to find the metabolites whose content was significantly influenced by the growing year, the variety, and the cropping system, respectively. All metabolites indicated by PCA as related to year- and variety-associated variability (Table 5) showed significant variations according to ANOVA results. Moreover, significant interactions “year–variety” and “year–cropping system” were found for a major part of the metabolites. For example, proline levels showed both year- and variety-associated differences, as well as a year–variety interaction. Proline content significantly increased in the second year, but mostly for the “O” variety, see Figure S1. Independent of the cultivation system, the proline level was higher for the “C” variety than for the “O” variety in the first year, whereas the opposite trend was observed for the second year.

**Figure 6:**
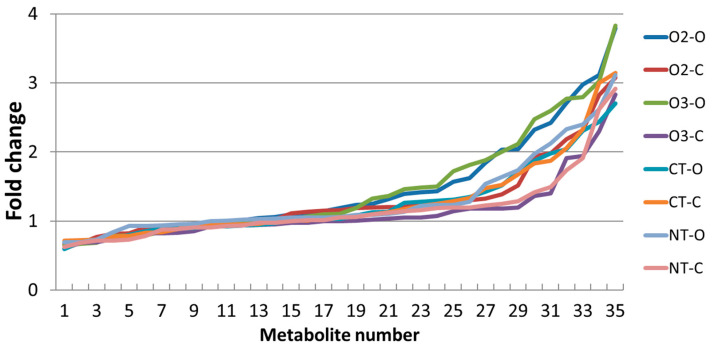
Fold changes (FC) (second year/first year ratio) for eight combinations of the cropping system (O2, O3, CT, and NT) and the variety (organic “O” or conventional “C”) labeled by different colors. The FC values were calculated using the mean value for 35 metabolites and were ordered from t lowest to highest.

Although ANOVA indicated that the levels of 19 out of 35 metabolites were significantly different under different cultivation conditions (column S of Table 5), none of them can be considered as a marker of the cropping system due to strong interactions with the year factor. These interactions indicate a strict synergistic relationship between environmental conditions and agronomic practices as factors influencing the metabolome. This could be exploited for mitigation of natural impacts, such as adverse climatic conditions. In order to explore this possibility, the variation in the metabolite levels between the two years for each combination of the cropping system (O2, O3, CT, and NT) and the variety (organic or conventional) was summarized and compared by calculating fold changes (FC) (second year/first year) for each metabolite (Figure 6). For comparison, the FC values were ordered from lowest to highest (see Table S1 for details). As can be seen in Figure 6, the levels of a major part of the metabolites for all combinations were higher in the second year, but clear differences between cropping system/variety combinations were also observed. The lowest positive FC values were observed for two combinations: NT or O3 and the conventional variety, whereas O2 or O3 combinations with the organic variety showed the highest positive FC values.

**Figure 7:**
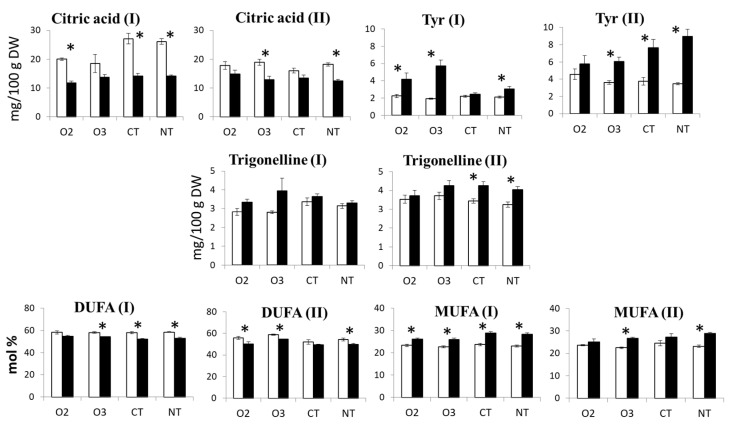
Box plots with means and standard errors of selected metabolites extracted from the maize grain. White and black boxes correspond to “O” and “C” varieties, respectively. The (I) and (II) correspond to the 1st and the 2nd year, respectively. Asterisks indicate the significant difference (*p* < 0.05) between “O” and “C” means.

Finally, taking into account PCA and ANOVA, a few metabolites (Figure 7) can be selected as markers of variety independently of year-to-year variability. The levels of citric acid and diunsaturated fatty acids (DUFA) were higher in the organic variety compared to the conventional variety, whereas tyrosine, trigonelline, and monounsaturated fatty acids (MUFA) were higher in the conventional variety compared to the organic variety.

## 3. Discussion

The present study aimed at characterizing the composition and nutritional value of maize grain as influenced by the cropping system, the genetic background, and the growing year using untargeted NMR metabolomics. NMR is known to be a robust and high-throughput untargeted analytical approach, enabling a comprehensive picture of the metabolite profile to reveal the main sources of metabolite variation [21]. NMR profiling of water-soluble metabolites has already been used to compare GM and non-GM maize grains [24] and to investigate the genetic basis of complex plant traits in a wide range of maize hybrids [28].

### 3.1. Metabolite Profile

#### 3.1.1. Amino Acids

The free amino acid content of maize grains has previously been determined not only by NMR [28] but also by HPLC [5], therefore giving the possibility for cross-validation of different analytical methods. In contrast to HPLC, in the present study it was not possible to determine the content of some amino acids such as Arg, Glu, Gln, Gly, Lys, and His by quantitative NMR analysis. Unfortunately, in a complex mixture such as the maize grain extract, ^1^H NMR spectral resolution was limited by signal splitting due to spin–spin couplings and overlap of the ^1^H NMR signal of a given metabolite with the signals of other molecules. Asparagine, aspartic acid, and proline were the most abundant free amino acids (Table 2), as previously reported for other studies [5,28]. However, the mean values of Pro, Asn, and Asp content (35.22, 20.96, and 10.59 mg/100g DW, respectively) were lower in comparison to the literature data [5,28] (Pro, 140–40 mg/100 g DW; Asn, 60–25 mg/100 g DW; and Asp, 40–23 mg/100 g DW). It is noteworthy that the variability of the proline content was the highest among the amino acids. In addition, proline is known to be an osmoprotectant, and its accumulation in maize seedlings [29] subjected to osmotic stress has been reported. The content of the remaining less-abundant amino acids (Ala, GABA, Ile, and Val) was also lower with respect to the literature data [5,28], except for the aromatic amino acids (Phe and Tyr), whose content was more or less comparable with previous studies.

#### 3.1.2. Sugars

The content of sucrose, the most abundant sugar in the maize grain extracts, was in agreement with previously reported data [28]. The high relative levels of sucrose, derived from leaf tissue, can be explained by its biological role as a building block used by maize kernels for starch synthesis. Trisaccharide raffinose, the second most abundant sugar after sucrose, is well known for its antinutrient properties, being non-digestible by humans and causing flatulence upon bacterial fermentation in the large intestine. The level of raffinose showed modest variation due to the genetic background and the growing year. On the contrary, the glucose level was highly variable and quite low with respect to the literature (mean 53.53 mg/100g DW in comparison to 100–180 mg/100g DW [28]. Traces of fructose and galactose were observed in the NMR spectra but were not quantified as they were below the limits of quantification.

#### 3.1.3. Organic Acids

In accordance with the literature [5], citric acid was found to be the most abundant organic acid in the maize grain. Compared to the reported data [28], the content of the less abundant malic, acetic, and lactic acids was significantly lower. For comparison, the mean values of malic acid in several hybrid lines were in the range of 40–90 mg/100 g DW, about 10–20 times higher than observed here. In addition, there was a very high degree of variation in the malic acid content (>152 fold). Formic acid and succinic acid showed the lowest levels, consistent with previously reported data [28].

#### 3.1.4. Fatty Acids/Lipids

The relative content of the four different types of fatty acids (separated according to the number of double bonds in the chain) was generally in agreement with the literature [5], except for PUFA (linolenic acid), whose content was too low in our case (0.54% vs. 1.1–1.2% in the literature [5]), and SFA (stearic and palmitic acids), whose content was slightly high (20% vs. 13% [5]). According to the literature, β-sitosterol and campesterol are the most abundant sterols in corn oil, and stigmasterol content is 10–12 times lower with respect to the sum of β-sitosterol and campesterol [30]. In our case, the mean content of stigmasterol was 24 times lower than the sum of β-sitosterol and campesterol; that is quite consistent with the literature. No literature data on lipid composition (mono-, di-, and triglycerides, PC) were available for comparison.

#### 3.1.5. Miscellaneous Metabolites

Free glycerol detected in maize grain and derived probably from triacylglycerides hydrolysis is often considered to be an important osmolyte in many organisms [31]. The total glycerol content measured here showed extensive variability (>59-fold), and the mean value (99.92 mg/100 g DW, Table 2) was substantially higher compared to the literature (10–14 mg/100 g DW) [5]. The choline and trigonelline levels were consistent with reported data [28], while no data have been reported for glycerophosphorylcholine.

### 3.2. Effect of Variety, Cropping System, and Growing Year on Metabolite Profile

The present study expands the set of metabolites, complementing the previous study where phenolic acids, flavonoids, some organic acids, and sugars were analyzed by UHPLC coupled with mass spectrometry (MS) [22]. Here, a different multivariate statistical analysis approach was applied that consisted of a combination of explorative unsupervised analysis (PCA) and ANOVA instead of variance decomposition analysis. In spite of the different sets of metabolites, the different extraction and analytical methods, and the distinct statistical methodologies employed, the cumulative findings of the present and preceding studies [22] exhibit a notable degree of consistency. The two studies share the following results:

- The most significant component of the explainable variance was the random effect due to the naturally occurring year-related impact;
- Among the fixed factors (the variety and the cropping system), the maize variety was the most significant factor contributing to the overall variability;
- The effect of the cropping system was overwhelmed by the random effect of the growing year;
- A substantial proportion of the total variance was unexplained.

First, the importance of the year-related variability was demonstrated by PCA, where the first most important principal component (PC1) responsible for net separation of the samples of the first and the second year accounted for 23.4% of the total variability. Moreover, according to ANOVA, 30 out of 35 metabolites showed significant mean differences according to year or year/variety or year/cropping system interactions. It is worth mentioning that previous studies based on gas chromatography/mass spectrometry metabolite profiling methodologies [32,33] have shown that the growing year has a bigger impact on the maize grain metabolome than the cropping system, the variety, or the location. As can be inferred from PCA, the first four principal components account for 61.4% of the total variability that comprises the variability due to the year, the variety, and the cropping systems, whereas the remaining 38.6% is related to unexplained variability probably due to considerable biological variability in the system, as reported in the previous study [22].

A significant influence of variety on maize grain metabolome has been demonstrated in a number of studies [5,28,32,33]. For example, in a wide range of maize hybrid varieties significant differences in the levels of almost all amino acids, sugars (glucose, fructose, and sucrose) and organic acids (fumaric acid, malic acid, and succinic acid) were observed [28]. A prevalence of variety-associated impact over cropping system impact (conventional versus organic farming) on metabolite profiles of maize grain has been observed [32]. Only three metabolites (malic acid, *myo*-inositol, and phosphate) out of 169 identified metabolites were consistently different because of the employed cropping system.

As previously stated, it was not feasible to ascertain the net impact of the cropping system due to the significant interplay between this factor and the year factor. On the one hand, this is a clear obstacle for the development of effective agronomic practices aimed at enhancing the nutritional quality of maize grain. On the other hand, the interaction between environmental conditions and agronomic practices as factors influencing the metabolome can be exploited for the mitigation of natural impacts, such as adverse climatic conditions. The potential consequences of climate changes due to global warming represent a significant and growing concern within the agricultural production industry, and many efforts have been implemented with the aim of addressing this challenge [28,32]. Our results demonstrate that the year-related natural impact on the metabolome varied between the different cropping systems, indicating that the organic cultivation systems were the most susceptible to the year-related variations, whereas the combination of the conventional variety with the conventional NT or organic cropping systems (O3) was the most resilient to the year-related variations. However, as long as it is not really understood what caused the variations in metabolites, the observed trends may be a consequence of other factors not related to climate, and therefore further more detailed studies are needed to support these conclusions.

## 4. Materials and Methods

### 4.1. Maize Grain Samples

Maize was cultivated in the field site, long-term USDA-ARS Farming Systems Project (FSP) at the Beltsville Agricultural Research Center, Beltsville, MD, USA (39.0° N, 76.9° W), as previously described [22]. The FSP plots were established in 1996 in a randomized complete block split-plot design with cropping system as the main plot, crop as the split, and with four replicates. Two of the cropping systems are managed using conventional (a three-year conventional no-till rotation (NT) and a three-year conventional chisel-till rotation (CT)), and two using organic management practices (a two-year organic rotation (Org2) and a three-year organic rotation (Org3)) [23,34]. Maize variety TA 65713 VP was used in conventional systems and Blue River variety 53R57 was used in organic systems.

Maize grain samples of conventional and organic varieties grown side-by-side within four FSP cropping systems were sampled for this study, as previously described [22]. Totally, 48 samples were collected during 2012 (2 varieties, 4 cropping systems, and 6 field replicates), and 64 samples were collected in 2013 (2 varieties, 4 cropping systems, and 8 field replicates). Corn grains were lyophilized and ground to obtain a fine powder and stored at −20 °C until extractions.

### 4.2. Sample Preparation

#### 4.2.1. Extraction Procedure

A 2:1 *v*/*v* mixture of methanol and chloroform (1.5 mL) was added to 150 mg of corn grain powder and stirred for 30 s, followed by the addition of chloroform (0.5 mL) and distilled water (0.9 mL). After the addition of each solvent, the sample was stirred again and finally centrifuged at 12,000 rpm (5000× *g*) for 5 min. The upper hydroalcoholic phase and the lower chloroform phase were separated from the pellet. The extraction was repeated with the pellet and the same amounts of solvents. The separated liquid phases from the 1st and 2nd extractions were combined. The solvents were evaporated at room temperature with N_2_ flow.

#### 4.2.2. NMR Sample Preparation

Dried residues were dissolved in 0.7 mL of D_2_O/phosphate buffer (hydroalcoholic extracts) or CDCl_3_ (chloroform extracts) and transferred to standard 5 mm NMR glass tubes. The D_2_O/phosphate buffer contained 400 mM solution of phosphate at pH = 7 and 1 mM of 3-(trimethylsilyl)propionic-2,2,3,3-d_4_ acid sodium salt (TSP) as an internal standard.

### 4.3. NMR Spectra Acquisition and Processing

The NMR spectra of hydroalcoholic and organic extracts were recorded at 27 °C on a Bruker AVANCE 600 NMR spectrometer (Bruker BioSpin GmbH, Rheinstetten, DE, USA) operating at the proton frequency of 600.13 MHz. Proton spectra were referenced to the methyl group signal of TSP (δ = 0.00 ppm) in D_2_O, and the methyl group signal of tetramethylsilane (δ = 0.00 ppm) added in CDCl_3_. Proton spectra of aqueous extracts were acquired by coadding 256 transients with a recycle delay of 7 s. The residual HDO signal was suppressed by presaturation. The experiment was carried out using a 90° pulse of approximately 14.0 μs, 32 K data points. Proton spectra of CDCl_3_ extracts were obtained using the following parameters: 256 transients, 64 K data points, recycle delay of 3 s, and a 90° pulse of 9–10 μs. After zero filling to 64 K data points and Fourier transformation using exponential multiplication factor (LB, 0.3 Hz), the phase and baseline corrections were carried out manually.

Two-dimensional NMR experiments, namely ^1^H-^1^H TOCSY, ^1^H-^13^C HSQC, and ^1^H-^13^C HMBC, were performed for metabolite identification and spectral assignment. The mixing time for the TOCSY was 80 ms. The HSQC experiments were performed using a coupling constant ^1^*J*_C–H_ of 150 Hz, and the HMBC experiments were performed using a delay for the evolution of long-range couplings of 80 ms.

### 4.4. Quantitative NMR Analysis

#### 4.4.1. Hydroalcoholic Extracts

Water-soluble metabolites were quantified by the integration of selected signals (Table 1) and normalization of the integrals in regard to those of three methyl groups of TSP at 0.0 ppm used as internal standard. The weight of selected metabolites (mg/100 g of dry seed powder) was calculated from the molar concentrations obtained from the integrals.

#### 4.4.2. Chloroform Extracts

For CDCl_3_ spectra, the integrals of 13 selected signals labeled with numbers (I_1_–I_13_) were measured (see Table 2). The integrals were normalized with regard to the integral of α-CH_2_ groups of all fatty acid chains (the sum of esterified (I_5_) and free (I_6_) fatty acid chains was set to 100%). Then, the total content (% mol) of fatty acids of four different types (saturated fatty acids (SFA), monounsaturated fatty acids (MUFA), diunsaturated fatty acids (DUFA), and polyunsaturated fatty acids (PUFA)) were calculated according to Equations (1)–(4). The content of PC, 1-MG, 2-MG, 1,2-DG, 1,3-DG, and TG (in % mol) were also calculated as the molar% of corresponding lipid multiplied by the number of esterified fatty acid chains in each molecule (one for 1-MG and 2-MG; two for PC, 1,2-DG, and 1,3-DG; and three in the case of TG).

PUFA = 2·I_3_/1.5, (1)

DUFA = I_7_ − 4·I_3_/1.5, (2)

MUFA = I_4_/2 − DUFA − PUFA, (3)

SFA = 100 − MUFA − DUFA − PUFA,(4)

### 4.5. Statistical Analyses

Principal component analysis (PCA) and analysis of variance (ANOVA) followed by post hoc comparison were carried out using Statistica 6.0 software package for Windows (Statsoft).

## Figures and Tables

**Table 1:** Summary of water-soluble metabolites identified in 600 MHz ^1^H NMR spectra of maize grain extracts in D_2_O/phosphate buffer.

Compound	Assignment	^1^H, (ppm)	Multiplicity: *J*_H-H_ [Hz]
Organic acids			
Acetic acid	CH_3_	1.92	s
Citric acid	α,γ-CH	2.54	d [15.1]
Formic acid	CH	8.46	s
Lactic acid	β-CH_3_	1.33	d [6.9]
Malic acid	CH_2_	2.37	dd [15.4; 10.0]
Succinic acid	CH_2_	2.41	s
Carbohydrates			
α-Glucose	CH-1	5.24	d [3.7]
β-Glucose	CH-1	4.66	d [8.0]
Raffinose	CH-1	5.00	d [3.7]
Sucrose	CH-4′	4.06	t [8.6]
Amino acids			
Alanine (Ala)	β-CH_3_	1.49	d [7.3]
Asparagine (Asn)	β,β′-CH_2_	2.89	dd [16.9; 7.3]
Aspartic acid (Asp)	β,β′-CH_2_	2.82	dd [17.4; 3.8]
γ-aminobutyrate (GABA)	α-CH_2_	2.30	t [7.5]
Isoleucine (Ile)	γ′-CH_3_	1.01	d [7.0]
Phenylalanine (Phe)	CH-3,5 (ring)	7.43	t [7.4]
Proline (Pro)	γ-CH_2_	2.00	m
Tyrosine (Tyr)	CH-2,6 (ring)	7.20	d [8.4]
Valine (Val)	γ′-CH_3_	1.05	d [7.0]
Miscellaneous metabolites			
Choline	N(CH_3_)_3_	3.21	s
Glycerophosphorylcholine	N(CH_3_)_3_	3.23	s
Glycerol	CH_2_	3.64	dd [11.7; 4.3]
Trigonelline	CH	8.85	s

**Table 2:** Mean, maximum, and minimum values of the metabolite content (mg/100g DW) of the water-soluble extracts of the maize grain.

Metabolite	Mean	Maximum	Minimum	Max/Min
	Organic acids			
Acetic acid	3.81	6.72	1.98	3.4
Citric acid	16.69	33.76	5.62	6.0
Formic acid	2.31	7.63	1.07	7.1
Lactic acid	1.40	3.13	0.45	6.9
Malic acid	4.91	13.69	0.09	152.0
Succinic acid	2.20	5.75	0.53	10.9
	Carbohydrates			
Glucose	53.53	165.35	13.22	12.5
Raffinose	153.83	249.74	88.90	2.8
Sucrose	1641.96	2119.21	1256.28	1.7
	Amino acids			
Ala	6.24	9.06	2.88	3.1
Asn	20.96	32.51	10.04	3.2
Asp	10.59	16.77	2.28	7.4
GABA	1.46	3.20	0.45	7.1
Ile	0.53	1.56	0.20	7.9
Phe	1.59	3.83	0.56	6.9
Pro	35.22	70.66	7.04	10.0
Tyr	4.41	12.06	1.69	7.2
Val	1.42	2.66	0.73	3.6
	Miscellaneous metabolites			
Choline	6.71	12.17	2.96	4.1
Glycerophosphorylcholine	12.35	29.30	6.82	4.3
Glycerol	99.92	273.71	16.55	16.5
Trigonelline	3.57	7.17	2.38	3.0

**Table 3:** Summary of metabolites identified in 600 MHz ^1^H spectra of maize grain extracts in CDCl_3_.

Compound	Assignment ^1^	Integral Label	^1^H, (ppm)	Multiplicity: *J*_H-H_ [Hz]
Campesterol + *β*-Sitosterol (CS)	**CH_3_**	I_1_	0.68	s
Stigmasterol (ST)	**CH_3_**	I_2_	0.70	s
Linolenic acid	**CH_3_**	I_3_	0.98	t
All unsaturated fatty acids	**CH_2_**-CH=CH	I_4_	2.05–2.01	m
Esterified fatty acids (EFA)	**CH_2_**-COOR	I_5_	2.31	m
Free fatty acids (FFA)	**CH_2_**-COOH	I_6_	2.35	t
Di- and triunsaturated fatty acids	CH=CH-**CH_2_**-CH=CH	I_7_	2.81–2.77	t
Phosphatidylcholine (PC)	N(**CH_3_**)_3_	I_8_	3.29–3.26	s
1-Monoacylglyceride (1-MG)	3-**CH_2_**OH	I_9_	3.60	dd [11.5; 5.8]
2-Monoacylglyceride (2-MG)	1,3-**CH_2_**OH	I_10_	3.84	d [4.6]
1,3-Diacylglyceride (1,3-DG)	1,3-**CH_2_**OR	I_11_	4.18	m
1,2-Diacylglyceride (1,2-DG)	1-**CH_2_**OR	I_12_	4.24	dd [12.0; 5.7]
Triacylglyceride (TG)	1,3-**CH_2_**OR	I_13_	4.29	dd [12.0; 4.3]

^1^ The functional group that gives the selected NMR signal is highlighted in bold characters.

**Table 4:** Mean, maximum, and minimum values of the metabolite content (mol%) of chloroform extracts of the maize grain.

Metabolite ^1^	Mean	Maximum	Minimum	Max/Min
CS	1.20	1.40	1.03	1.4
ST	0.05	0.08	0.04	2.1
PUFA	0.54	0.72	0.25	2.8
DUFA	54.26	62.54	37.31	1.7
MUFA	25.25	31.37	15.95	2.0
SFA	19.95	32.01	13.35	2.4
FFA	46.09	78.88	26.46	3.0
PC	1.43	2.03	0.50	4.0
1-MG	2.52	5.41	0.81	6.7
2-MG	0.10	0.24	0.03	7.2
1,3-DG	3.40	5.00	1.91	2.6
1,2-DG	1.31	1.92	0.74	2.6
TG	52.17	75.92	17.50	4.3

^1^ Abbreviations: Campesterol + *β*-Sitosterol (CS); Stigmasterol (ST); polyunsaturated fatty acids (PUFA) diunsaturated fatty acids (DUFA); monounsaturated fatty acids (MUFA); saturated fatty acids (SFA); free fatty acids (FFA); Phosphatidylcholine (PC); 1-Monoacylglyceride (1-MG); 2-Monoacylglyceride (2-MG); 1,3-Diacylglyceride (1,3-DG); 1,2-Diacylglyceride (1,2-DG); Triacylglyceride (TG).

**Table 5:** Three-way ANOVA results. Abbreviations: Y, growing year; V, variety; S, cropping system, YxV, YxS, VxS, YxVxS interactions between factors. Asterisks indicate the significant difference between the means (* 0.01 < *p* < 0.05; ** 0.001 < *p* < 0.01; *** *p* < 0.001).

Metabolite	Y	V	S	YxV	YxS	VxS	YxVxS
Acetic acid	*						
Citric acid	***	***		***	***		*
Formic acid	***		**		*		
Lactic acid					*		
Malic acid		***	**	*	**		
Succinic acid	***	*	*				
Glucose	***	***	*	***	**		
Raffinose		***		***			
Sucrose	***	***	***	*	***		
Ala	***			**	*		
Asn				***	*		*
Asp	***		***				*
GABA	***		***		***		
Ile	***			***			*
Phe	***			***	*		
Pro	***	*	***	***	***		
Tyr	***	***		*	**		***
Val	***		**	***			**
Choline	***		*	**			
GPCholine		*	**				
Glycerol	**		**		***		
Trigonelline	***	***					
CS		***		***			
ST		*	**				
PUFA		*		**			
DUFA	***	***	**		*		
MUFA		***	**			*	
SFA	***				*		
FFA	**		***		*		
PC		*	*		**		
1-MG							
2-MG							
1,3-DG	***						
1,2-DG	***		**				
TG	***		***		***		

## Data Availability

The raw data supporting the conclusions of this article will be made available by the authors on request.

## Supplementary Materials

The following supporting information can be downloaded at: https://www.mdpi.com/article/10.3390/molecules29174097/s1, Figure S1: Histograms of mean values and standard errors of water-soluble metabolites extracted from maize grain; Figure S2: Histograms of mean values and standard errors of liposoluble metabolites extracted from maize grain; Table S1: Fold changes of mean values.
