# Supplementary material for: Maize Grain Metabolite Profiling by NMR: Effects of Growing Year, Variety, and Cropping System
Source: Molecules. 2024 Aug 29;29(17):4097. doi: 10.3390/molecules29174097 (PMC11397060; doi:10.3390/molecules29174097)
Supplement: Supplementary file 1 [file molecules-29-04097-s001.zip › molecules-3146064-supplementary.pdf]

Supplementary materials for

Maize grain metabolite profiling by NMR: Effects of growing year, variety and cropping system

Anatoly Petrovich Sobolev[1,*],Erica Acciaro[1], Milica Milutinović[2], Jelena Božunović[2], Neda Aničić[2], Danijela Mišić[2] and Autar K Mattoo[3,*]

1 Magnetic Resonance Laboratory "Annalaura Segre", Institute for Biological Systems, National Research Council (CNR), via Salaria km 29.300, 00015 Monterotondo, Rome, Italy;
2 Institute for Biological Research "Siniša Stanković"-National Institute of Republic of Serbia, University of Belgrade, Belgrade, Serbia
3 Beltsville Agricultural Research Center, Beltsville, MD, USA
* Correspondence: anatoly.sobolev@cnr.i (A.P.S.); Autar.Mattoo@USDA.gov (A.K.M.)

Table of Content

Figure S1. 2

Figure S2. 4

Table S1. 5

Figure S1: Histograms of mean values and standard errors of water-soluble metabolites extracted from maize grain. White and black boxes correspond to "O" and "C" varieties, respectively. The (I) and (II) correspond to the 1st and the 2nd year, respectively.

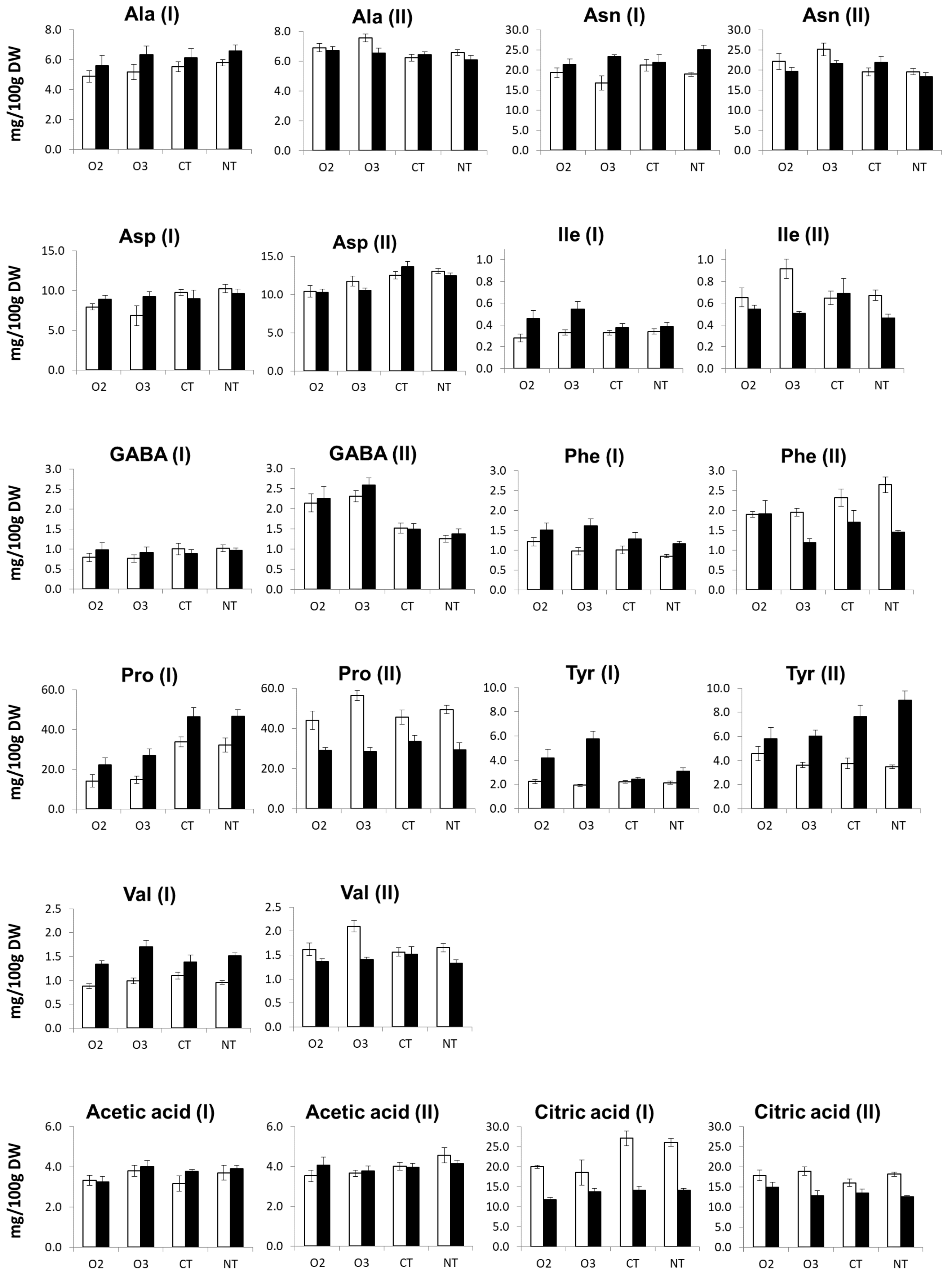

Figure S1 continue

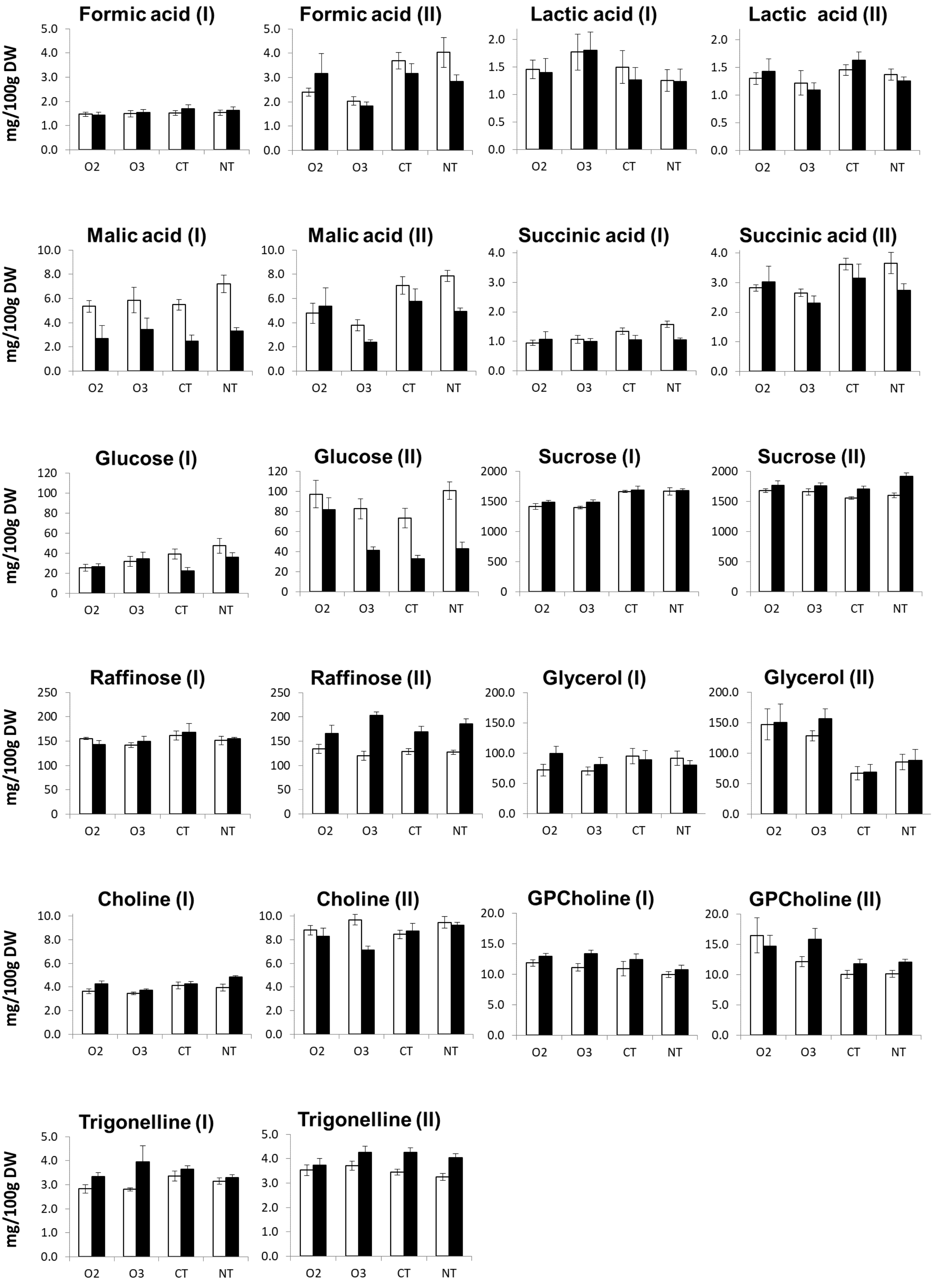

Figure S2: Histograms of mean values and standard errors of liposoluble metabolites extracted from maize grain. White and black boxes correspond to "O" and "C" varieties, respectively. The (I) and (II) correspond to the 1st and the 2nd year, respectively.

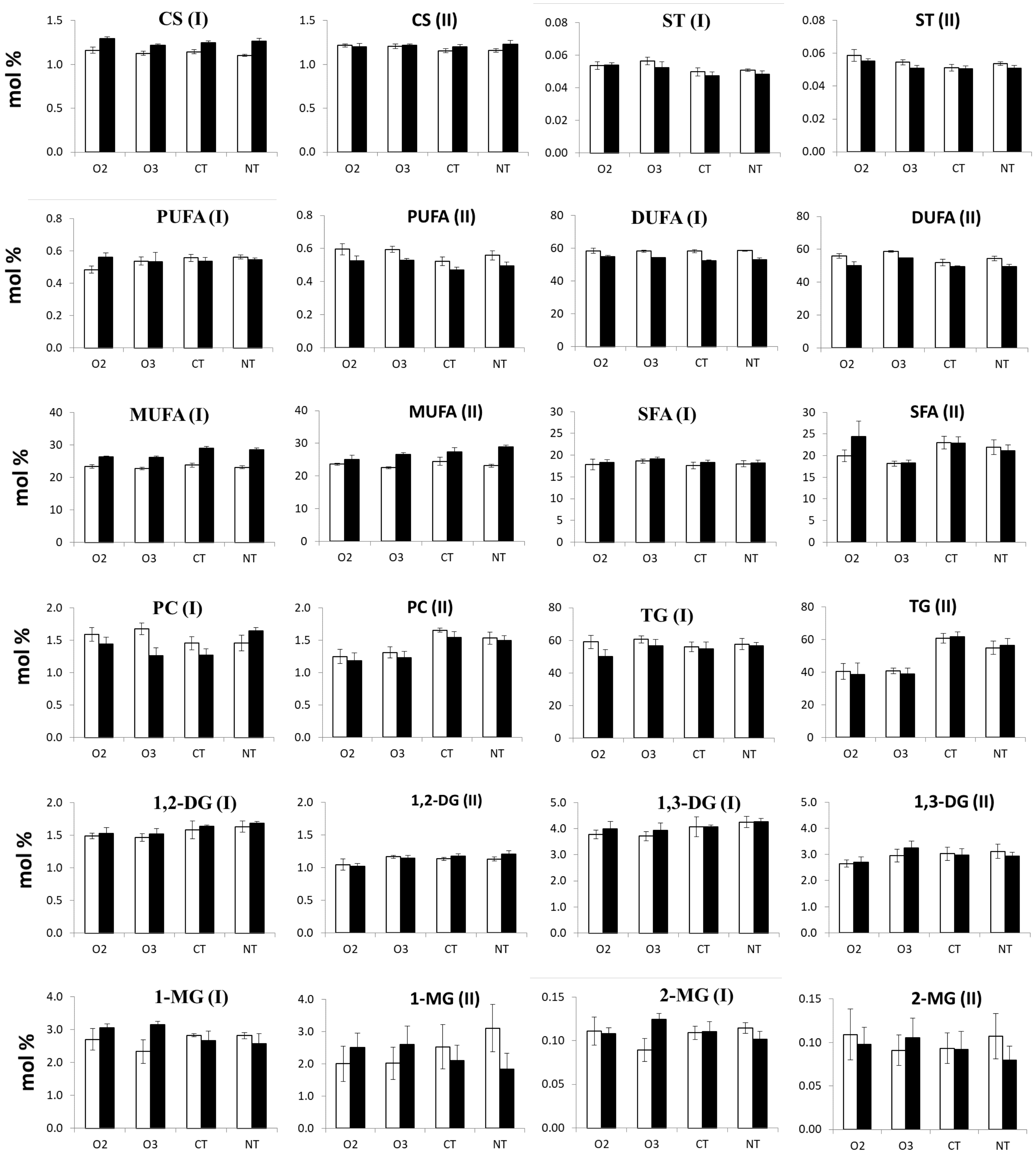

Table S1: Fold changes of mean values (second year/first year) calculated and ordered from the lowest to the highest for each combination of cropping system (O2, O3, CT, NT) and variety (O vs C).

O2-O		O2-C		O3-O		O3-C	
Metabolite	FC	Metabolite	FC	Metabolite	FC	Metabolite	FC
TG	0.69	1,2 -DG	0.67	Malic A	0.65	Lactic A	0.61
1,2 -DG	0.70	1,3-DG	0.68	TG	0.67	TG	0.69
1,3-DG	0.70	TG	0.77	Lactic A	0.69	Malic A	0.70
1-MG	0.74	PC	0.82	PC	0.78	Phe	0.74
PC	0.78	1-MG	0.82	1,2 -DG	0.79	1,2 -DG	0.75
Raffinose	0.86	2-MG	0.90	1,3-DG	0.80	1,3-DG	0.82
Lactic A	0.89	DUFA	0.91	Raffinose	0.84	1-MG	0.82
Malic A	0.89	Asn	0.92	1-MG	0.87	Val	0.83
CITRIC A	0.89	CS	0.93	AcA	0.96	2-MG	0.85
DUFA	0.96	PUFA	0.94	ST	0.97	Asn	0.92
2-MG	0.98	MUFA	0.96	SFA	0.97	Ile	0.93
MUFA	1.01	Val	1.01	MUFA	0.99	CITRIC A	0.94
CS	1.05	Lactic A	1.02	DUFA	1.01	AcA	0.94
AcA	1.06	ST	1.02	CITRIC A	1.02	SFA	0.96
ST	1.10	TRIG	1.11	2-MG	1.02	ST	0.98
SFA	1.12	GPCholine	1.13	CS	1.07	PC	0.98
Asn	1.14	Asp	1.15	GPCholine	1.10	PUFA	1.00
Sucrose	1.19	Raffinose	1.16	PUFA	1.11	CS	1.00
PUFA	1.23	Ile	1.19	Sucrose	1.18	DUFA	1.01
TRIG	1.25	Sucrose	1.19	TRIG	1.32	MUFA	1.02
Asp	1.32	FFA	1.20	Formic A	1.36	Ala	1.03
GPCholine	1.39	Ala	1.20	Ala	1.46	Tyr	1.05
Ala	1.41	AcA	1.26	FFA	1.49	Pro	1.05
FFA	1.43	CITRIC A	1.26	Asn	1.50	TRIG	1.08
Phe	1.57	Phe	1.28	Asp	1.72	Asp	1.14
Formic A	1.62	Pro	1.30	Glycerol	1.81	Formic A	1.18
Val	1.84	SFA	1.33	Tyr	1.88	Sucrose	1.18
Glycerol	2.04	Tyr	1.38	Phe	2.00	GPCholine	1.18
Tyr	2.04	Glycerol	1.51	Val	2.12	Glucose	1.20
Ile	2.33	Choline	1.94	Succinic A	2.48	Raffinose	1.36
Choline	2.42	Malic A	1.98	Glucose	2.60	FFA	1.40
GABA	2.71	Formic A	2.19	Ile	2.77	Choline	1.91
Succinic A	2.98	GABA	2.31	Choline	2.80	Glycerol	1.94
Pro	3.12	Succinic A	2.82	GABA	3.02	Succinic A	2.30
Glucose	3.79	Glucose	3.08	Pro	3.83	GABA	2.83
CT-O		CT-C		NT-O		NT-C	
Metabolite	FC	Metabolite	FC	Metabolite	FC	Metabolite	FC
CITRIC A	0.59	1,2 -DG	0.72	1,2 -DG	0.69	Pro	0.63
Glycerol	0.70	Pro	0.72	CITRIC A	0.70	1,3-DG	0.69
1,2 -DG	0.72	1,3-DG	0.73	1,3-DG	0.73	1,2 -DG	0.71

1,3-DG	0.74	Glycerol	0.78	Raffinose	0.84	1-MG	0.72
Raffinose	0.80	1-MG	0.79	DUFA	0.93	Asn	0.73
2-MG	0.86	FFA	0.83	Glycerol	0.93	2-MG	0.78
1-MG	0.89	2-MG	0.83	2-MG	0.94	Val	0.87
DUFA	0.89	PUFA	0.88	TG	0.95	CITRIC A	0.88
FFA	0.90	MUFA	0.94	Sucrose	0.96	PUFA	0.91
GPCholine	0.92	DUFA	0.95	PUFA	0.99	PC	0.91
Asn	0.92	CITRIC A	0.95	MUFA	1.01	Ala	0.93
Sucrose	0.94	GPCholine	0.96	GPCholine	1.02	DUFA	0.94
PUFA	0.94	CS	0.96	Asn	1.03	CS	0.98
Lactic A	0.97	Asn	1.00	TRIG	1.03	FFA	0.98
CS	1.01	Raffinose	1.00	PC	1.05	TG	1.00
TRIG	1.02	Sucrose	1.01	CS	1.05	MUFA	1.01
ST	1.03	AcA	1.05	FFA	1.05	Lactic A	1.02
MUFA	1.03	Ala	1.06	ST	1.06	ST	1.05
TG	1.08	ST	1.07	Malic A	1.09	AcA	1.06
Ala	1.13	Val	1.09	Lactic A	1.09	Glycerol	1.10
PC	1.14	TG	1.13	1-MG	1.10	GPCholine	1.12
AcA	1.27	TRIG	1.17	Ala	1.14	Sucrose	1.14
Asp	1.28	PC	1.22	SFA	1.22	SFA	1.16
Malic A	1.29	SFA	1.25	AcA	1.23	Glucose	1.19
SFA	1.31	Lactic A	1.29	GABA	1.23	Ile	1.20
Pro	1.35	Phe	1.33	Asp	1.28	Raffinose	1.20
Val	1.43	Glucose	1.48	Pro	1.53	TRIG	1.22
GABA	1.52	Asp	1.52	Tyr	1.63	Phe	1.25
Tyr	1.71	GABA	1.68	Val	1.73	Asp	1.29
Glucose	1.87	Ile	1.84	Ile	1.97	GABA	1.42
Ile	1.98	Formic A	1.88	Glucose	2.12	Malic A	1.50
Choline	2.04	Choline	2.05	Succinic A	2.33	Formic A	1.74
Phe	2.31	Malic A	2.33	Choline	2.40	Choline	1.91
Formic A	2.44	Succinic A	3.00	Formic A	2.62	Succinic A	2.62
Succinic A	2.70	Tyr	3.14	Phe	3.11	Tyr	2.92
